# PMMA dialyzers modulate both humoral and cell-mediate immune response to anti-COVID-19 vaccine (BNT162b2) in a cohort of chronic hemodialyzed patients

**DOI:** 10.1038/s41598-024-62044-9

**Published:** 2024-05-28

**Authors:** Giuseppe Castellano, Giuseppe Stefano Netti, Vincenzo Cantaluppi, Vincenzo Losappio, Federica Spadaccino, Elena Ranieri, Marita Marengo, Maurizio Borzumati, Carlo Alfieri, Giovanni Stallone

**Affiliations:** 1 Nephrology, Dialysis and Renal Transplantation Unit, Fondazione IRCCS Cà Granda Ospedale Maggiore Policlinico, Via Commenda 15, 20122 Milan, Italy; 2 Department of Clinical Sciences and Community Health, University of Milan, Milan, Italy; 3 Unit of Clinical Pathology, Center for Molecular Medicine and Advanced Research Center on Kidney Aging (A.R.K.A.), Department of Medical and Surgical Sciences, University of Foggia, Foggia, Italy; 4 Unit of Nephrology and Kidney Transplantation, Department of Translational Medicine, University of Piemonte Orientale (UPO), 17-28100 Novara, Italy; 5 Unit of Nephrology Dialysis and Transplantation, Advanced Research Center on Kidney Aging (A.R.K.A.), Department of Medical and Surgical Sciences, University of Foggia, Foggia, Italy; 6 Unit of Nephrology and Dialysis, Azienda Sanitaria Locale (ASL) CN1, Cuneo, Italy; 7 Unit of Nephrology and Dialysis, ASL VCO, Verbania, Verbano Cusio Ossola Italy

**Keywords:** SARS-CoV-2 vaccination, Hemodialysis patients, Poly-methyl-methacrylate, Vaccine antibody response, Humoral immunity, Cellular immunity, Haemodialysis, SARS-CoV-2

## Abstract

Patients on hemodialysis (HD) have a high risk of death from COVID-19. We evaluated the humoral and cell-mediated immune response to BNT162b2 (Pfizer-BioNTech) vaccine in HD patients, comparing HD with Poly-methyl-methacrylate (PMMA) and HD with Polysulphone (PS). Samples were collected before vaccination (T0) and 14-days after the 2ndvaccine (T2) in a TG (TG, n = 16-Foggia) and in a VG (CG, n = 36-Novara). Anti-SARS-CoV-2-Ig were titrated in the cohort 2-weeks after the 2nddose of vaccine. In the Testing-Group, serum neutralizing antibodies (NAb) were assayed and PBMCs isolated from patients were thawed, counted and stimulated with SARS-CoV-2 IGRA stimulation tube set. All patients had a positive ab-response, except in a case. PMMA-patients had higher levels of anti-SARS-CoV-2 IgG (*p* = 0.031); VG data confirmed these findings (*p* < 0.05). NAb evaluation: PMMA patients passed the positive cut-off value, while in PS group only only 1/8 patient did not respond. PMMA patients showed higher percentages of anti-SARS-CoV-2 S1/RBD-Ig after a complete vaccine schedule (*p* = 0.028). Interferon-gamma release: PMMA patients showed significantly higher release of IFNγ (*p* = 0.014). The full vaccination course provided sufficient protection against SARS-CoV-2 across the entire cohort, regardless of dialyzer type. After vaccination, PMMA patients show a better immune response, both humoral and cellular, at the end of the vaccination course than PS patients.

## Introduction

Patients on maintenance hemodialysis (HD) are at higher risk of infection with severe acute respiratory syndrome coronavirus 2 (SARS-CoV-2) and death from Coronavirus disease 2019 (COVID-19)^1,2^.

International guidelines recommend the priority of SARS-CoV-2 vaccination in this cohort of frail patients^3^. Immunological senescence and lack of response to vaccination are frequently observed in HD patients. Indeed, end stage renal disease (ESRD) patients develop an altered immune response to infection or vaccination, which may be affected by several factors, such as a decreased renal clearance, increased generation of pro-inflammatory cytokines, and mainly in the hemodialysis setting, over-hydration, poor dialyzer membrane biocompatibility, anticoagulation and vascular inflammation^4–7^.

The pro-inflammatory milieu in HD is associated with alterations in both the innate and adaptive immune response^8,9^.

The effects of dialysis membranes on vaccine response are not well known to date. It has been reported that the membranes for hemodialysis are involved in the chronic activation and dysregulation of the immune system, by affecting the reduction of Th2 and regulatory T cells function and by interfering their interaction with B lymphocyte by CD40/CD40L. Due to the presence of both pro-inflammatory status and immune response alterations in HD patients, high biocompatibility membranes such as polymethylmethacrylate (PMMA) have been introduced. These dialytic membranes are able to reduce the activation of complement system, coagulation, platelets and leukocytes, thus lowering the levels of pro-inflammatory cytokines such as IL1B, TNF-α, IL-6. Moreover, PMMA membranes exert a significant adsorptive effect to remove high molecular weight uraemic toxins, as compared to standard dialyzers such as Polysulphone (PS)^10,11^.

Messenger RNA (mRNA) vaccines, such as BNT162b2 (Pfizer-BioNTech) and mRNA-1273 (Moderna), and the replication-defective vial-vectored vaccines, such as ChAdOx1 nCOV-19 (Oxford-AstraZeneca), are considered low risk of complications in patients on maintenance dialysis^12^. In this study we evaluated the humoral and cell-mediate immune response to BNT162b2 (Pfizer-BioNTech) vaccine in patients in stable hemodialysis, treated with PMMA-based adsorptive HD or with conventional Polysulphone-based standard HD.

## Results

A cohort of 16 patients undergoing standard hemodialysis at the Nephrology Unit in Foggia (Italy) (*Testing Group*, TG) were enrolled in the present study, among which 8 were treated with adsorptive HD (AHD) and 8 were treated with standard HD (SHD) from at least 12 months. As shown in Table 1, both groups did not differ in the main clinical and laboratory parameters at baseline.

**Table 1:** Clinical and laboratory characteristics at baseline of HD patients enrolled in the study as Testing Group (n = 16).

	Standard HD (PS-based)	Adsorptive HD (PMMA-based)	*p*-value
Patients (n)	8	8	
Female gender (%)	12.5%	25.0%	*0.522*
Age (years)	60.3 ± 16.33	62.6 ± 9.79	*0.912*
Dialysis time (months)	45.4 ± 15.8	54.3 ± 25.4	*0.149*
Body weight (kg)	77.25 ± 20.7	80.75 ± 12.8	*0.478*
BMI (kg/m^2^)	25.75 ± 4.26	27.6 ± 3	*0.422*
Vascular Access, AVF (%)	100.0%	87.5%	*0.302*
KT/V	1.44 ± 0.28	1.36 ± 0.14	*0.457*
Hemoglobin (g/dL)	10.5 ± 1.4	11.7 ± 0.8	*0.192*
Leucocyte count (× 10^3^/mcl)	7.82 ± 2.55	8.37 ± 1.21	*0.444*
Albumin (g/dL)	3.67 ± 0.35	3.75 ± 0.22	*0.894*
Ca (mg/dL)	8.92 ± 0.72	8.99 ± 0.35	*0.783*
P (mg/dL)	4.81 ± 0.63	4.56 ± 0.59	*0.211*
PTH (pg/mL)	187.87 ± 75.03	192.37 ± 79.82	*0.497*
Ferritin (mcg/L)	99.5 ± 58.8	130.5 ± 125.3	*0.053*
Previous nephropathies			
*Diabetes*	2	2	
*Nephroangiosclerosis*	1	1	
*Vascular diseases*	0	1	
*ADPKD*	2	1	
*Glomerulopathies*	1	1	
*Others/Unknown*	2	2	

ADPKD, Autosomal dominant Polycistic Kidney disease; AVF, Arteriovenous fistula; BMI, body mass index; Polymethylmethacrylate, PMMA; PS, polysulphone.

Data are reported as percentage or as mean ± SD.

**Figure 1:**
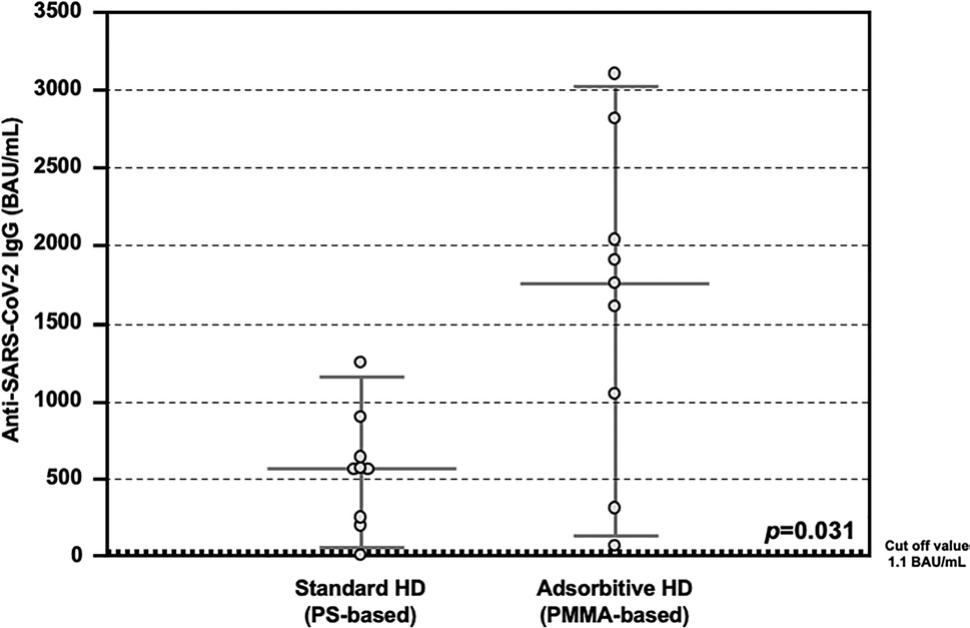
Anti-SARS-CoV-2 antibody response after COVID-19 mRNA vaccine in the Testing Group. Detection of total anti-SARS-CoV-2 IgG showing higher serum levels in patients treated with Adsorptive HD (AHD, n = 8), as compared to those treated with Standard HD (SHD, n = 8) (1755.0 IQR 864.3–2225.0 vs 566.0 IQR 237.8–704.0 BAU/mL, p = 0.031).

All the patients completed the vaccine schedule and 14 days after the administration of the second doses, the total anti SARS-CoV-2 IgG titer was assessed in the entire cohort. A positive antibody response was observed in all the hemodialyzed patients, except in a case. After stratification according to type of HD treatment, patients treated with AHD showed significantly higher levels of anti-SARS-CoV-2 IgG, as compared to those treated with SHD (1755.0 interquartile range or IQR 864.3–2225.0 vs 566.0 IQR 237.8–704.0 BAU/mL, *p* = 0.031; Fig. 1).

**Figure 2:**
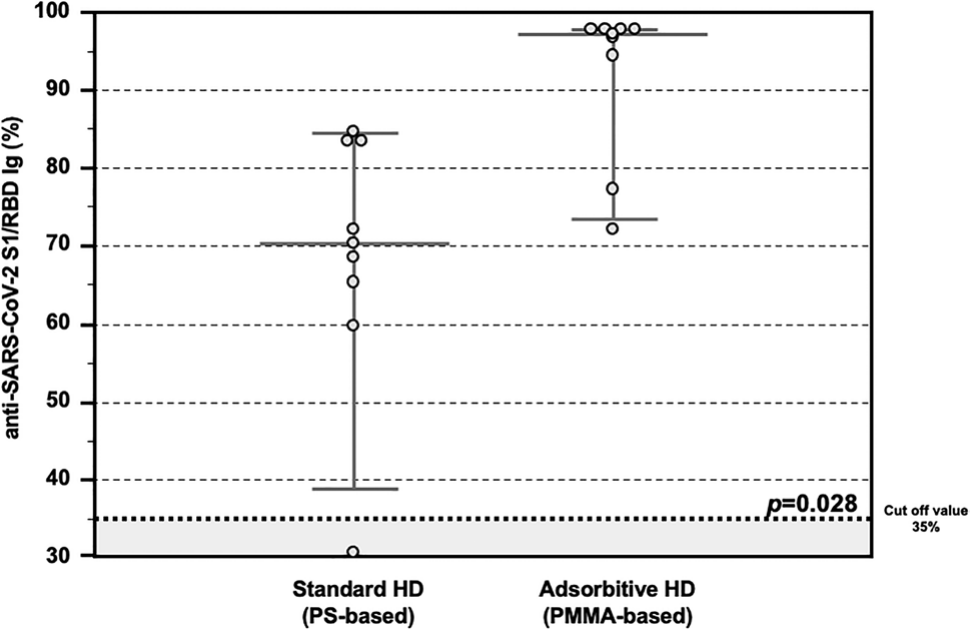
Anti-SARS-CoV-2 antibody response after COVID-19 mRNA vaccine in the Testing Group. Detection of total anti-SARS-CoV-2 S1/RBD Ig showing higher percentages in HD patients treated with Adsorptive HD (AHD, n = 8), as compared with those treated with Standard HD (SHD, n = 8) (97.3 IQR 90.2–97.8% vs 70.3 IQR 63.9–83.5%, p = 0.028). Data are shown as dots and whiskers (median and 95% CI).

Concerning the presence of neutralizing antibodies (NAb), the detection of serum anti-SARS-CoV-2 S1/RBD Ig titer was assessed with an enzyme-linked immunosorbent assay (ELISA)-based surrogate virus neutralization test (sVNT) and all the values above the manufacturer's specified cut-off value of 35% were considered as positive. In our *Testing Group* cohort, all patients treated with AHD passed the positive cut-off value, while among the patients treated with SHD only only 1/8 patient did not respond. Moreover, patients treated with AHD showed higher percentages of anti-SARS-CoV-2 S1/RBD Ig after a complete vaccine schedule, as compared with those treated with SHD (97.3 IQR 90.2–97.8% vs 70.3 IQR 63.9–83.5%, *p* = 0.028; Fig. 2). A significant positive correlation was observed between both assays (R^2^ = 0.5391, *p* < 0.001).

**Figure 3:**
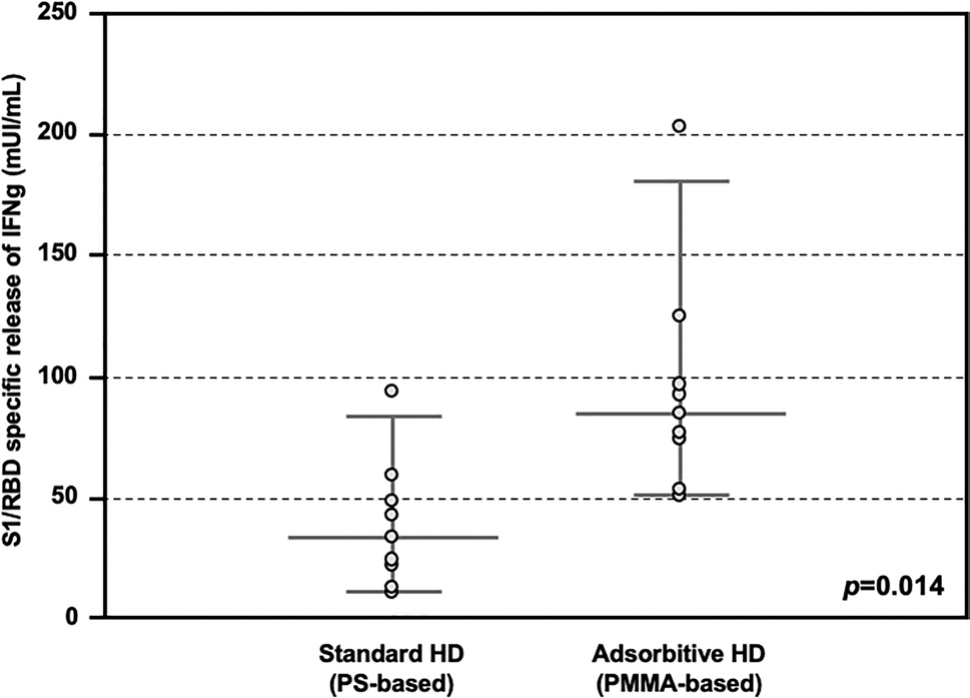
S1/RDB-specific IFNγ release response after COVID-19 vaccine in the Testing Group. Release of IFNγ from PBMC stimulated with SARS-CoV-2 S1/RBD, showing higher titer in patients treated with Adsorptive HD (AHD, n = 8), as compared with these treated with Standard HD (SHD, n = 8) (84.8 IQR 68.7–103.8 vs 33.5 IQR 19.7–51.1 mUI/mL, p = 0.014).

Then, the T cell response against COVID vaccine was also assessed. In detail, peripheral blood mononuclear cells isolated from both HD patients treated with AHD and SHD were tested with a SARS-CoV-2 interferon gamma (IFNγ) release assay (IGRA). HD patients treated with AHD showed significantly higher release of IFNγ, as compared with these treated with SHD (84.8 IQR 68.7–103.8 vs 33.5 IQR 19.7–51.1 mUI/mL, *p* = 0.014; Fig. 3).

**Figure 4:**
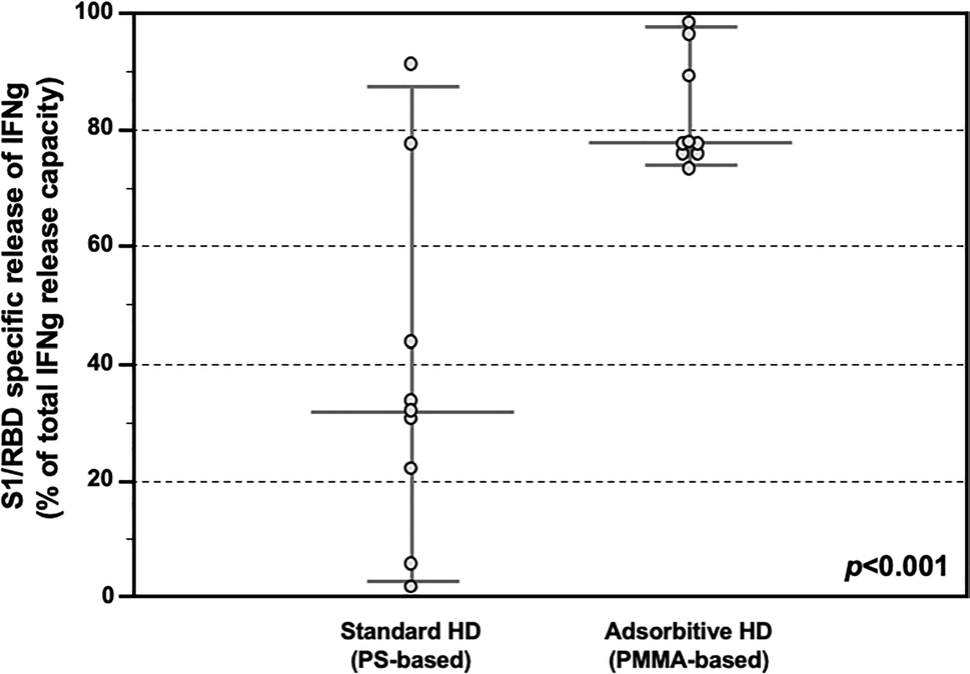
S1/RDB-specific IFNγ release response after COVID-19 vaccine in the Testing Group. Release of IFNγ from PBMC stimulated with SARS-CoV-2 S1/RBD, showing higher ratio (IFNγ released after SARS-CoV-2-related S1/RBD specific stimulus/ IFNγ release after aspecific mitogen exposure) in HD patients treated with Adsorptive HD (AHD, n = 8), as compared HD patients treated with Standard HD (SHD, n = 8) (77.9 IQR 76.1–91.1% vs 32.2 IQR 17.8–52.0% , p < 0.001). Data are shown as dots and whiskers (median and 95% CI).

Moreover, the T-cell reactivity was assessed as a ratio (IFNγ released after SARS-CoV-2-related S1/RBD specific stimulus/ IFNγ release after non-specific mitogen exposure). Also, in this case HD patients treated with AHD showed stronger capacity (%) to release IFNγ after specific stimulus as compared to the maximum release induced by non-specific mitogen, while this ratio was significantly lower in HD patients treated with SHD (77.9 IQR 76.1–91.1% vs 32.2 IQR 17.8–52.0%, *p* < 0.001; Fig. 4).

To validate our preliminary data about the possible role of adsorptive dialytic treatments in modulating immune response to COVID vaccine, we recruited a *Validation group* (VG) of 36 hemodialyzed patients at the Nephrology Unit of Novara (Italy). In this cohort of patients, 18 were treated with adsorptive HD (AHD) and 18 were treated with standard HD (SHD) from at least 12 months.

Both groups did not differ in the main clinical and laboratory parameters at baseline (Age 66.0 ± 13.0 vs 67.3 ± 11.4 years, Female Gender 23% vs 45%, dialysis vintage 36.8 ± 26.8 vs 40.0 ± 29.0 for AHD and SHD, respectively; *p* = ns) (Table 2).

**Table 2:** Clinical and laboratory characteristics at baseline of HD patients enrolled in the study as *Validation Group* (n = 36).

	Standard HD (PS-based)	Adsorptive HD (PMMA-based)	*p*-value
Patients (n)	18	18	
Female gender (%)	44.4%	22.2%	*0.157*
Age (years)	67.3 ± 11.4	66.0 ± 13.0	*0.897*
Dialysis time (months)	40.0 ± 29.0	36.8 ± 26.8	*0.657*
Body weight (kg)	78.15 ± 22.35	79.55 ± 15.65	*0.512*
BMI (kg/m^2^)	26.25 ± 3.20	27.15 ± 3.75	*0.875*
Vascular access, AVF (%)	83.3%	88.9%	*0.630*
KT/V	1.48 ± 0.31	1.39 ± 0.28	*0.475*
Hemoglobin (g/dL)	10.2 ± 1.7	11.1 ± 0.9	*0.127*
Leucocyte count (10^3^/mcl)	7.98 ± 2.35	8.95 ± 1.84	*0.493*
Albumin (g/dL)	3.52 ± 0.15	3.82 ± 0.31	*0.619*
Ca (mg/dL)	8.67 ± 0.71	8.94 ± 0.42	*0.717*
P (mg/dL)	5.12 ± 0.93	4.92 ± 0.87	*0.821*
PTH (pg/mL)	202.12 ± 65.10	197.81 ± 72.34	*0.368*
Ferritin (mcg/L)	110.5 ± 59.8	124.5 ± 98.6	*0.059*
Previous nephropathies			
*Diabetes*	4	4	
*Nephroangiosclerosis*	4	3	
*Tubulo-interstitial disease*	4	4	
*Vascular diseases*	1	0	
*ADPKD*	2	2	
*Glomerulopathies*	1	1	
*Others/Unknown*	2	4	

*ADPKD* Autosomal dominant Polycistic Kidney disease, *AVF* Arteriovenous fistula, *BMI* body mass index, *PMMA* Polymethylmethacrylate, *PS* polysulphone.

Data are reported as percentage or as mean ± SD.

**Figure 5:**
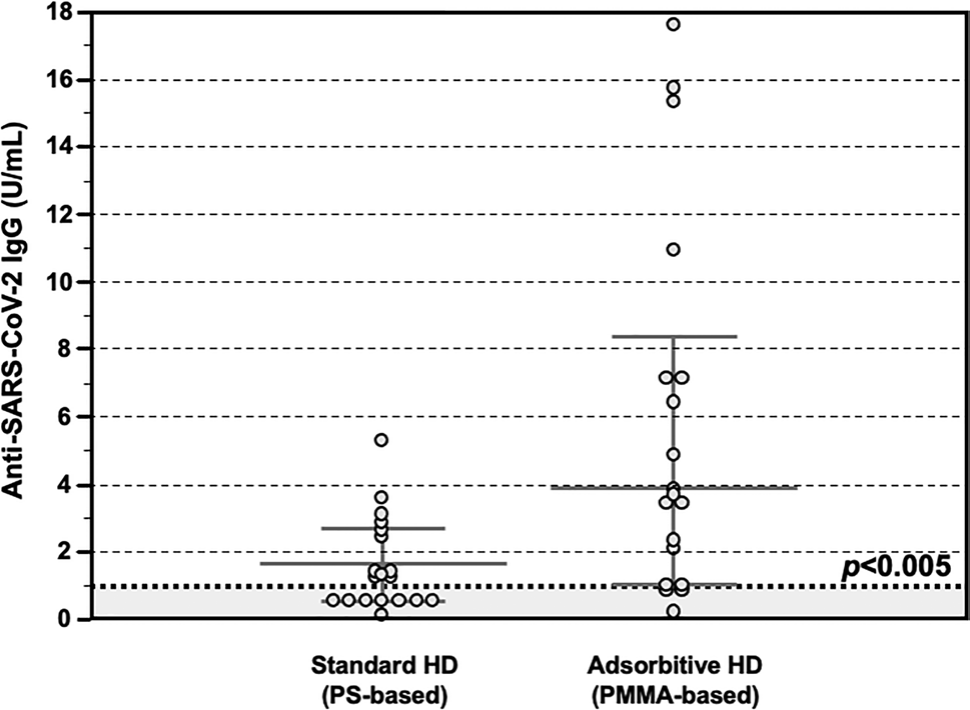
Anti-SARS-CoV-2 antibody response after COVID-19 mRNA vaccine in the *Validation Group*. Detection of total anti-SARS-CoV-2 IgG showing significantly higher serum levels in patients treated with Adsorptive HD (AHD, n = 18), as compared to those treated with Standard HD (SHD, n = 18) (4.9 IQR 3.5–6.9 vs 1.4 IQR 0.8–2.2 U/ml, p < 0.05). Data are shown as dots and whiskers (median and 95% CI).

Fourteen days after completing the 2-doses vaccination schedule, the total anti SARS-CoV-2 IgG titer was assessed in the entire cohort. Also in this group of patients being treated at a different dialysis center, those treated with AHD showed significantly higher levels of anti-SARS-CoV-2 IgG, as compared to those treated with SHD (4.9 IQR 3.5–6.9 vs 1.4 IQR 0.8–2.2 U/ml, *p* < 0.005) (Fig. 5).

## Discussion

In this report we showed for the first time that after vaccination course with BNT162b2 mRNA vaccine, HD patients under AHD with PMMA dialyzers show a better immune response, both humoral and cellular, than HD patients treated with SHD.

The severity of COVID-19 is linked to a significant influx of neutrophils and macrophages into the lungs, triggering an intense immune response and a cascade of severe molecular events resulting in systemic inflammation and multi-organ failure^13^. IL-6 stands out among the elevated serum molecules during cytokine storm, underscoring its crucial role^14–16^. The duration of seroconversion after Coronavirus infection in chronic dialysis patients^17^. Recent data also show that memory B and T cells are often maintained following viral infection. In fact, memory B cells specific for the virus spike protein were observed in the patients who had the infection and seem remain stable during the first 5 months.

However, virus-specific CD4 + T cells were detected in COVID-19-recovered individuals and correlated with plasma levels of S-specific antibodies. These findings confirm the reliance of memory B cell responses, a crucial aspect of long-lasting immunity against SARS-CoV-2 infection, on CD4 + T cells during COVID-19^18^.

Patients with ESRD and on HD, have innate and acquired immune response deficiency^19,20^. A diminished antibody production to thymo-dependent with conserved a response to thymo-independent antigens, shows an impairment of the co-stimulation of B cells by T-cell receptor-activated T lymphocytes, whereas the direct activation of B cells without the help of T lymphocytes is preserved^21,22^.

Several studies have shown that, although most dialysis membranes, including high-flux polysulfone, are unable to clear sCD40, polymethylmethacrylate (PMMA) membranes, above all BK-F membrane, allows a dramatic diminution of the molecule, so improving immune response in these patients and consequently the long-term response to hepatitis B vaccination^23,24^.

Clearing sCD40 from HD patient’s sera by using PMMA dialyzers contributes to the amelioration of the seroconversion rate to HBV vaccination in ESRD patients who failed to mount a protective immune response, improving their capacity to respond to HBV vaccination^25^.

Our study suggests that PMMA could enhance this serological response such as it has been observed in HD patients after HBV vaccination according to its ability to modulate sCD40 serological levels^26,27^. At the same time, recent evidences of failed serological immunity in HD patients not responding to HBV vaccination suggest that seroconversion may not be directly related to immunocompetence in patients with inflammaging: comparable functional HBsAg-reactive B and T cells responses has been proved in HD patients after HBV vaccination^28^ not only in the absence of a proper IgG serological response but even in the condition of reduced HBs-reactive Th2 cells^29^. These add a new piece to the proper evaluation of a proper immune response after mRNA-based vaccination protocols and the need to support immune response with additional doses only considering serological titers^30^.

Unfortunately, we did not dose sCD40 levels in our patients in order to evaluate a possible correlation between immunological response and its titers in AHD and SHD patient. At the same time our evidences suggest a possible direction for our further evaluation and the necessity to assess immune response with flow cytometry next to serological response. It is important to remember that one possible limit of the present study could be the fact that Anti-SARS-CoV-2 Ig was measured in the T and C groups using two different methods. These two methods are in any case validated and used currently in clinical practice.

In conclusion, the complete vaccination course conferred adequate protection against the SARS-CoV-2 in all patients observed in our study, either those on SHD or AHD, without any adverse events thus suggesting efficacy and safety of the BNT162b2 mRNA vaccine. This is the crucial and important message with important clincal significance. In addition it is important to underscore that HD patients under AHD with PMMA dialyzers show a better immune response, both humoral and cellular, at the end of the vaccination course than PS patients. The results presented in the present study could open future studies aimed to encompass dialyzers effects and their modulative effects in immuno-senescence HD patients.

## Methods

### Study population

The prospective observational and cohort study was performed including two groups of patients with ESRD undergoing replacement therapy with hemodialysis (HD) from two different Nephrology Units of the Southern and Norther Italy. The first group of 16 HD patients was enrolled at the Nephrology Dialysis and Transplantation Unit of the University Hospital "Ospedali Riuniti", Foggia (Italy) and was used as main cohort (*Testing Group*). The second group of 36 HD patients was enrolled at the Nephrology Dialysis and Transplantation Unit of the University of Eastern Piedmont “Amedeo Avogadro”, Novara (Italy) and was used to validate the results obtained in the testing group of patients (*Validation Group*).

All the enrolled patients were > 18 and < 80 years old and COVID-19 naïve. Exclusion criteria were: therapy with immunosuppressive drugs, previous kidney transplant, systemic infections, cancer, HIV positivity or other life-threatening conditions with life expectancy lower than 6 months.

This study aimed to compare the immunomodulatory effects of adsorptive dialytic treatments (Poly-methyl-methacrylate or PMMA-based) on COVID vaccine response compared to conventional Polysulphone-based dialytic treatments. Nevertheless, a limited number of patient chronically treated with PMMA membranes entering the inclusion criteria were followed at two different Nephrology Units involved in the study (26 HD patients). For this reason, a propensity-score matching analysis was conducted in R using the MatchIt package with nearest-neighbor 1-to-1 matching to compare HD patients treated with PMMA with HD patients treated with conventional dialytic membrane (Polysulfone).

All the enrolled patients were treated thrice a week with hemodialysis (HD) treatment for at least 12 months. Both in the *Testing Group [TG]* and in the *Validation Group [VG]*, half of the patients were treated with maintenance PMMA-based HD (AdsorptiveAdsorptive HD or AHD; n = 8 for *[TG]* and n = 18 for *[VG]*, respectively) and the remaining with maintenance conventional HD with Polysulphone (PS) (Standard HD or SHD; n = 8 for *[TG]* and n = 18 for *[VG]*, respectively).

Low-molecular weight or unfractionated heparin was administered as standard anti-coagulation therapy. Dialysis prescription was guided aiming at a value of urea reduction rate ≥ 0.65 and a Kt/V ≥ 1.2. The above parameters of dialysis adequacy were calculated according to the second-generation Daugirdas equation^31^.

To rule out the possibility of active or previous SARS-CoV-2 infection, all the patients of both groups were assessed for both PCR nasal swab and detection of anti-SARS-CoV-2 IgM and IgG, both resulted negative, and were therefore considered as SARS-CoV-2 naïve.

The two study groups were enrolled from each separate hospital. Several meetings before the study initiation were made to confirm the absence of difference on dialysis condition (water quality or COVID19 infection status) between the two dialytic centers.

After signing an informed consent to participate to the present study, all the enrolled patients received two doses of the anti-SARS-CoV-2 mRNA BNT16b2 Vaccine (Comirnaty, Pfizer-Biontech, USA). All the clinical data at enrolment were collected and recorded.

The study protocol conformed to the ethical guidelines of the Declaration of Helsinki and was approved by the institutional review board (Decision no. 1570/2021 of 04 April 2021; Ethical Committee at the University Hospital "Policlinico Riuniti" of Foggia). This was in accordance with the guidelines laid down by the Regional Ethics Committee on human experimentation.

### Sample collection

In all the enrolled subjects of both the groups, serum samples were collected before vaccination (Time 0, T0) and fourteen days after the second vaccine dose (Time 2, T2) and stored at − 30 °C, until analyzed.

Only in the enrolled subjects of the *Testing Group*, whole blood (25 ml) was collected from all patients at T0 and T2, as previously described^32^. Peripheral blood mononuclear cells (PBMCs) were isolated by density separation on SepMateTM (STEMCELL Technologies, Vancouver, Canada), according to manufacturer’s instructions, and stored at − 80 °C, until analyzed.

### Detection of anti SARS-CoV-2 antibodies

Anti-SARS-CoV-2 Ig were titrated in both *Testing Group* and *Validation Group* two week after the second dose of vaccine with different methods, according to the local laboratory protocols.

In detail, as previously described^32^, anti-SARS-CoV-2 IgG and IgM titre in the *Testing Group* was analyzed by using a chemiluminescent analytical assay (CLIA) commercially available kit (New Industries Biomedical Engineering Co., Ltd [Snibe], Shenzhen, China), according to the manufacturer instructions. Reagent wells were coated with recombinant structural protein CoV-S (spike) and e CoV-N (nucleocapside) of SARS-CoV-2 for both IgM and IgG assay. For IgM assay, the microspheres were coated with a monoclonal antibody to capture human IgM followed by the addition of recombinant antigen from virus 2019-nCoV marked with amino-butylethyl-isoluminol (ABEI). The samples, serum or plasma, were diluted by instrument. The relative light units (RLU) detected was proportional to the concentration of IgG/M in sample. An RLU-ratio of the measurement of each sample to the supplied calibrator was calculated. According to manufacturer instructions, IgG assay BAU/mL of < 1 was considered negative, 1.0–1.1 borderline and > 1.1 positive; for IgM, an BAU/mL < 0.9 was considered negative, 0.9 to 1.0 borderline and > 1.0 positive. Clinical sensitivity was estimated by the manufacturer as 78.65% and 91.21% for IgM and IgG, respectively, while specificity was estimated as 97.50% and 97.3% for IgM and IgG, respectively.

Anti-SARS-CoV-2 IgG titre in the *Validation Group* was analyzed with a quantitative method for detection of IgG antibodies against the S1-RBD antigen (Atellica IM SARS-CoV-2 IgG [sCOV2G], Siemens Healthineers, Erlangen, Germany). This test is a fully automated, 2-step sandwich immunoassay, with indirect chemiluminescent technology. The patient specimen is incubated with preformed complex of streptavidin-coated particles and biotinylated SARS-CoV-2 recombinant antigens. The antibody-antigen complex is detected by an acridinium ester–labeled antihuman IgG mouse mAb. According to manufacturer instruction, IgG assay > 1.00 was considered positive. Clinical sensitivity for IgG was estimated by the manufacturer as 96.41%, while specificity was estimated as 99.90%.

### Neutralizing antibodies levels assessment

Only in the enrolled subjects of the *Testing Group*, serum neutralizing antibodies (NAb) levels were assayed, using a commercially available ELISA Kit, according to the manufacturer’s instructions (SARS-CoV-2 NeutraLISA, EUROIMMUN Medizinische Labor diagnostika AG, Lübeck, Germany), as previously described^32^. This competitive semi-quantitative test allows to evaluate the ability of Nab to prevent the link between the S1/RBD domain and the ACE2 receptor. In detail, microplate was coated with recombinant S1/RBD domain of SARS-CoV-2. Sample and controls were diluted 1:5 in dilution buffer containing soluble ACE2 conjugated to biotin and incubated in the reaction wells. Both Nab and soluble ACE2 competed for the binding site on the antigen surface. The photometric measurement at 450 nm yielded the results as a percentage of inhibition (%IH). According to manufacturer instructions, 20%IH was considered negative, 20 to 35%IH borderline and > 35%IH positive.

### Interferon-gamma release assay (IGRA)

Only in the enrolled subjects of the *Testing Group*, PBMCs isolated from patients were thawed, counted and stimulated with SARS-CoV-2 IGRA stimulation tube set (EUROIMMUN Medizinische Labor diagnostika AG, Lübeck, Germany), as previously described^32^.

In details, 1*106 PBMCs were resuspended in PBS/EDTA and dispensed in each of the three stimulation tubes for 20 h: CoV-2 IGRA BLANK for the determination of the background concentration of IFNy; CoV-2 IGRA STIM containing a mitogen causing non-specific secretion of IFNy; CoV-2 IGRA TUBE containing SARS-CoV-2 S1 components for the determination of specific IFNy secretion. After stimulation, samples were centrifuged and the supernatants used for subsequent quantitative assay using IFNy ELISA, according to the manufacturer instructions (EUROIMMUN Medizinische Labor diagnostika AG, Lübeck, Germany). Reaction wells were coated with anti-IFNy monoclonal antibody. Samples and controls were diluted 1:5 in a diluent buffer, incubated and processed according to manufacturer instructions. For IFNy quantification a 4-parameters logistics was applied.

### Statistical analysis

Statistical analysis was performed using SPSS 25.0 software (IBM Corp., Armonk, NY. Variable distribution was tested using Kolmogorov–Smirnov test. Serum parameters were compared between groups by Student’s *t*-test for unpaired data and Mann–Whitney *U*-test, as appropriate. Frequencies were compared among groups by F-Fisher or X2-test, as appropriate. Correlation between two variables was ascertained by Pearson or Spearman’s correlation tests, as appropriate. All the data are reported as mean ± standard deviation (SD), median and interquartile range (IQR), or as percentage frequency, unless otherwise specified. A *p*-value < 0.05 was considered statistically significant.

## Data availability

The dataset for this study may be made available upon request to the corresponding author.

## Abbreviations

HD: Hemodialysis
SARS-CoV-2: Coronavirus 2
COVID-19: Coronavirus disease 2019
ESRD: End stage renal disease
PMMA: Polymethylmethacrylate
PS: Polysulphone
mRNA: Messenger RNA
TG: Testing Group
VG: Validation Group
SHD: Standard HD
AHD: Adsorptive HD
Nab: Neutralizing antibodies
IFNγ: Interferon-gamma
Time 0, T0: Time before vaccination
Time 2, T2: Time after the second vaccine dose
PBMCs: Peripheral blood mononuclear cells
CLIA: Chemiluminescent analytical assay
ABEI: Amino-butylethyl-isoluminol
RLU: Relative light units
%IH: Percentage of inhibition
SD: Standard deviation

## References

[1] Grupper A, Sharon N, Finn T, Cohen R, Israel M, Agbaria A, Rechavi Y, Schwartz IF, Schwartz D (2021). Humoral response to the pfizer BNT162b2 vaccine in patients undergoing maintenance hemodialysis. Clin J Am Soc Nephrol..

[2] Taji L, Thomas D, Oliver MJ, Ip J, Tang Y, Yeung A (2021). COVID-19 in patients undergoing long-term dialysis in Ontario. CMAJ..

[3] Francis A, Baigent C, Ikizler TA, Cockwell P, Jha V (2021). The urgent need to vaccinate dialysis patients against severe acute respiratory syndrome coronavirus 2: A call to action. Kidney Int..

[4] Verkade MA, van de Wetering J, Klepper M, Vaessen LM, Weimar W (2004). Peripheral blood dendritic cells and GM-CSF as an adjuvant for hepatitis B vaccination in hemodialysis patients. Kidney Int..

[5] Kato S, Chmielewski M, Honda H, Pecoits-Filho R, Matsuo S (2008). Aspects of immune dysfunction in end-stage renal disease. Clin J Am Soc Nephrol..

[6] Betjes MG (2013). Immune cell dysfunction and inflammation in end-stage renal disease. Nat Rev Nephrol..

[7] Dai L, Golembiewska E, Lindholm B, Stenvinkel P (2017). End-stage renal disease, inflammation and cardiovascular outcomes. Contrib Nephrol..

[8] Meier P, Dayer E, Blanc E, Wauters JP (2002). Early T cell activation correlates with expression of apoptosis markers in patients with end-stage renal disease. J Am Soc Nephrol..

[9] Degiannis D, Moowat AM, Galloway E, Tsakiris D, Briggs JD (1987). In vitro analysis of B lymphocyte function in uraemia. Clin Exp Immunol..

[10] Losappio V, Franzin R, Infante B, Godeas G, Gesualdo L (2020). Molecular mechanisms of premature aging in hemodialysis: The complex interplay between innate and adaptive immune dysfunction. Int J Mol Sci..

[11] Zhang DL, Liu J, Cui WY, Ji DY, Zhang Y (2011). Differences in bio-incompatibility among four biocompatible dialyzer membranes using in maintenance hemodialysis patients. Ren Fail..

[12] Windpessl M, Bruchfeld A, Anders HJ, Kramer H, Waldman M (2021). COVID-19 vaccines and kidney disease. Nat Rev Nephrol..

[13] Behrens EM, Koretzky GA (2017). Review: Cytokine storm syndrome: looking toward the precision medicine era. Arthritis Rheumatol..

[14] Liu Y, Zhang C, Huang F, Yang Y, Wang F (2020). Elevated plasma levels of selective cytokines in COVID-19 patients reflect viral load and lung injury. Natl Sci Rev.

[15] Chen X, Zhao B, Qu Y, Chen Y, Xiong J (2020). Detectable serum severe acute respiratory syndrome coronavirus 2 viral load (RNAemia) Is closely correlated with drastically elevated interleukin 6 level in critically ill patients with coronavirus disease 2019. Clin Infect Dis..

[16] Vabret N, Britton GJ, Gruber C, Hegde S, Kim J (2020). Sinai immunology review project. Immunology of COVID-19: Current state of the science. Immunity..

[17] Seow J, Graham C, Merrick B, Acors S, Pickering S (2020). Longitudinal observation and decline of neutralizing antibody responses in the three months following SARS-CoV-2 infection in humans. Nat Microbiol..

[18] Grifoni A, Weiskopf D, Ramirez SI, Mateus J, Dan JM, Moderbacher CR, Rawlings SA, Sutherland A, Premkumar L, Jadi RS (2020). Targets of T cell responses to SARS-CoV-2 coronavirus in humans with COVID-19 disease and unexposed individuals. Cell..

[19] Higgins RM (1989). Infections in a Renal Unit. Q J Med..

[20] Contin-Bordes C, Lacraz A, de Précigout V (2010). Potential role of the soluble form of CD40 in deficient immunological function of dialysis patients: New findings of its amelioration using polymethylmethacrylate (PMMA) membrane. NDT Plus..

[21] Gaciong Z, Alexiewicz JM, Linker-Israeli M, Shulman IA, Pitts TO (1991). Inhibition of immunoglobulin production by parathyroid hormone. Implications in chronic renal failure. Kidney Int..

[22] Raskova J, Ghobrial I, Czerwinski DK, Shea SM, Eisinger RP (1987). B-cell activation and immunoregulation in end-stage renal disease patients receiving hemodialysis. Arch Intern Med..

[23] Bonomini M, Fiederling B, Bucciarelli T, Manfrini V, Di Ilio C (1996). A new polymethylmethacrylate membrane for hemodialysis. Int J Artif Organs..

[24] Cohen G, Rudnicki M, Schmaldienst S, Hörl WH (2002). Effect of dialysis on serum/plasma levels of free immunoglobulin light chains in end-stage renal disease patients. Nephrol Dial Transplant..

[25] Aoike I, Gejyo F, Arakawa M (1995). Learning from the Japanese Registry: How will we prevent long-term complications? Niigata Research Programme for beta 2-M Removal Membrane. Nephrol Dial Transplant..

[26] Stumpf J, Siepmann T, Lindner T, Karger C, Schwöbel J (2021). Humoral and cellular immunity to SARS-CoV-2 vaccination in renal transplant versus dialysis patients: A prospective, multicenter observational study using mRNA-1273 or BNT162b2 mRNA vaccine. Lancet Reg Health Eur..

[27] Olsen SK, Brown RS (2006). Hepatitis B treatment: lessons for the nephrologist. Kidney Int.

[28] Roch T, Giesecke-Thiel C, Blazquez-Navarro A, Wehler P, Thieme CJ (2021). Generation of HBsAg-reactive T- and B-cells following HBV vaccination in serological non-responders under hemodialysis treatment. Eur J Immunol..

[29] Awad G, Roch T, Stervbo U, Kaliszczyk S, Stittrich A (2021). Robust hepatitis B vaccine-reactive T cell responses in failed humoral immunity. Mol Ther Methods Clin Dev..

[30] Barda N, Dagan N, Cohen C, Hernán MA, Lipsitch M (2021). Effectiveness of a third dose of the BNT162b2 mRNA COVID-19 vaccine for preventing severe outcomes in Israel: An observational study. Lancet..

[31] Daugirdas JT (1993). Second generation logarithmic estimates of single-pool variable volume Kt/V: An analysis of error. J Am Soc Nephrol..

[32] Netti GS, Infante B, Troise D, Mercuri S, Panico M (2022). mTOR inhibitors improve both humoral and cellular response to SARS-CoV-2 messenger RNA BNT16b2 vaccine in kidney transplant recipients. Am J Transplant..

